# Development and Validation of a Prognostic Score for Hepatocellular Carcinoma Patients in Immune Checkpoint Inhibitors Therapies: The Hepatocellular Carcinoma Modified Gustave Roussy Immune Score

**DOI:** 10.3389/fphar.2021.819985

**Published:** 2022-02-08

**Authors:** Yongjiang Li, Yangxun Pan, Ximeng Lin, Jingyu Hou, Zili Hu, Li Xu, Zhongguo Zhou, Yaojun Zhang, Minshan Chen, Dandan Hu

**Affiliations:** ^1^ Collaborative Innovation Center for Cancer Medicine, State Key Laboratory of Oncology in South China, Sun Yat-Sen University Cancer Center, Guangzhou, China; ^2^ Department of Nuclear Medicine, Sun Yat-Sen University Cancer Center, Guangzhou, China; ^3^ Department of Liver Surgery, Sun Yat-Sen University Cancer Center, Guangzhou, China

**Keywords:** hepatocellular carcinoma, immune checkpoint inhibitors, Gustave Roussy immune score, lactate dehydrogenase, neutrophil-to-lymphocyte ratio, albumin, aspartate transaminase-to-alanine transaminase ratio, total bilirubin

## Abstract

**Background:** There is not yet an effective marker in predicting the efficacy of immune checkpoint inhibitors (ICIs) in treating hepatocellular carcinoma (HCC) patients. The Gustave Roussy Immune Score (GRIm-Score) based on three objective variables, namely, neutrophil-to-lymphocyte ratio (NLR), serum albumin level (ALB), and lactate dehydrogenase (LDH), was developed as feasible prognostic indication in lung cancer patients receiving ICIs therapies. Our study aimed to adapt the GRIm-Score (HCC-GRIm-Score) in HCC patients who received ICIs therapies and thus improving the predictive ability.

**Methods:** From January 2018 to September 2020, 261 patients who received ICIs therapy were retrospectively included and divided into training and validation groups. After determining the factors for HCC-GRIm-Score by multivariable analysis from training group, the optimized HCC-GRIm-Score was validated and compared to the original GRIm-Score and the Barcelona clinic liver cancer (BCLC) staging system.

**Results:** One hundred sixty-one and 80 patients were assigned into the training and validation groups, respectively. Two more factors, aspartate transaminase-to-alanine transaminase ratio [hazard ratio (HR), 1.51; 95% confidence interval (CI), 0.94–2.42] and total bilirubin (HR, 1.76; 95% CI, 1.07–2.88), were identified as independent prognostic factors for overall survival (OS) and integrated in the HCC-GRIm-Score system according to the multivariable analysis. A risk score based on the HCC-GRIm-Score indicated that patients presenting high score (>2) suffered from significantly shorter median OS of 10.3 months compared to those with a low score (not reached; HR, 2.99; 95% CI, 1.89–4.75; *p* < 0.001). In the validation group of 80 patients, the patients presenting a high score showed an inferior OS (HR 5.62, 95% CI, 1.25–25.24; *p* = 0.024). HCC-GRIm-Score had the highest area under curve of 0.719 (95% CI, 0.661–0.773) compared to original GRIm-Score and BCLC staging system.

**Conclusion:** The present study confirmed that the modified HCC-GRIm-Score system provided superior predictive ability in identifying the HCC patients potentially benefit from ICIs therapies, compared to the original GRIm-Score and the BCLC staging system.

## Introduction

Hepatocellular carcinoma (HCC) ranks as the sixth most common cancer and the third leading cause of cancer-related death worldwide in 2020 among 185 countries (Sung et al., 2021). In China, a chronic hepatitis B virus (HBV) high-prevalence infection area, the incidence and mortality rates of HCC are significantly higher than the average levels around the world (Cao et al., 2020). Given that the early symptoms of HCC are generally inconspicuous, most patients are diagnosed at an advanced stage, in which cases the curative-intended treatments are inapplicable. According to the clinical guidelines, targeted therapies have been the only systemic therapeutic agent available for HCC patients at an advanced stage for more than 10 years (Marrero et al., 2018; Zhou J et al., 2020; Llovet et al., 2021). Although recent global phase III studies reported promising results when adding regorafenib and lenvatinib to the medication of HCC, the improvement in the overall survival (OS) rate remained unsatisfactory (Bruix et al., 2017; Kudo et al., 2018).

During recent years, plenty of immune-modulatory agents were introduced for oncological treatment, eventually leading to the clinical breakthrough of immune checkpoint inhibitors (ICIs) targeting programmed death-1 (PD-1), programmed death-ligand 1 (PD-L1), or cytotoxic T-lymphocyte antigen-4 (CTLA-4) (Finn et al., 2020; Galle et al., 2021; Kelley et al., 2021). The emergence of ICIs further enriched the choices of oncologists in treating HCC patients who were unable to undergo curative treatments and resulted in convincing survival benefit in HCC patients who suffered from disease progression or unacceptable adverse effects with molecular targeted therapies. However, despite the definite survival benefit and the manageable toxicity profile that was proved in ICIs, the response rates of ICIs were disappointedly varying from 18.8 to 24.0% (Kudo et al., 2018; Kelley et al., 2021; Pan et al., 2021). Therefore, to maximally increase the anti-tumor efficacy among the patients who might potentially benefit from ICIs, the establishment of a prognostic and efficacy evaluation system could facilitate the individualized therapeutic strategy in clinical practice.

The Gustave Roussy Immune Score (GRIm-Score) developed by the Royal Marsden Hospital (RMH) prognostic score was initially validated in patients enrolled in the phase I trials with ICIs (Bigot et al., 2017). The original GRIm-Score (Ori-GRIm-Score) is a combination of neutrophil-to-lymphocyte ratio (NLR), serum albumin level (ALB), and lactate dehydrogenase (LDH) that stratifies patients in classes of high and low risk. In detail, patients with Ori-GRIm-Score >1 are considered as high risk for adverse prognosis. GRIm-Score presented a strong prognostic indication for patients toward immunotherapies, and its reliability has already been confirmed in several studies for lung cancer patients treated with cytotoxic chemotherapy, epidermal growth factor receptor-tyrosine kinase inhibitors (EGFR-TKI), or second-line immunotherapy (Minami et al., 2019; Lenci et al., 2021). However, the Ori-GRIm-Score has never been evaluated in a population based on HCC patients receiving ICIs, and its potential predictive value during ICIs treatment is yet to be investigated.

In the present study, based on the Ori-GRIm-Score, an HCC-modified GRIm-Score (HCC-GRIm-Score) for HCC patients who received ICIs was developed and validated according to the training and validation groups, respectively.

## Materials and Methods

### Patients

Patients who were diagnosed with HCC and received ICIs at Sun Yat-sen University Cancer Center (SYSUCC) between January 2018 and September 2020 were screened for eligibility. The inclusion criteria were as follows: (1) clinical diagnosis of HCC based on the high-risk factors, imaging characteristics, and serological molecular marker accourding to the guideline (Zhou J et al., 2020); (2) patients received at least two courses of ICIs treatment; (3) aged from 20 to 76; (4) sufficient liver function Child–Pugh (CP) stage of A or B; (5) absence of other malignant tumors; and (6) had complete medical and follow-up data. Exclusion criteria were (1) co-existence of other malignancies; (2) received ICIs other than PD-1, including PD-L1 and CTLA-4; and (3) incomplete follow-up data. The follow-up data, laboratory serological data, and imaging evaluation including enhanced computed tomography (CT) or magnetic resonance imaging (MRI) were extracted at the initial ICIs treatment.

The analysis of patient data was reviewed and approved by the Institutional Review Board and Human Ethics Committee at the SYSUCC (B2021-172-01) and was conducted consistent with the ethical guidelines of the 1975 Declaration of Helsinki.

### Treatments

ICIs were given and dissolved with saline in 30 min intravenously. ICIs are usually applied in the combination with transarterial interventional therapies or EGFR-TKI including sorafenib, lenvatinib, regorafenib, or apatinib during treatment. Patients who developed disease progression, unacceptable toxicity were considered to discontinue ICIs according to multidisciplinary consultation. The exact types and dosages of different ICIs are summarized in the Supplementary Table S1.

### Development of HCC-GRIm-Score

The Ori-GRIm-Score was a summation of NLR, LDH, and ALB, which was calculated as previously described by Bigot et al. (Bigot et al., 2017). In brief, patients were assigned 1 point if they had either NLR > 4.8, LDH > upper limit normal (ULN), or ALB < 35 g/L, for a total of three points. Ori-GRIm-Score <1 point was considered as a low score.

To develop a novel HCC-GRIm-Score based on the original one, the patients were divided into training and validation groups based on the time of starting ICIs therapies, respectively. Two new prognostic factors, aspartate transaminase-to-alanine transaminase ratio (AST-to-ALT ratio) and total bilirubin (TBIL), were identified according to multivariable analysis from the training group and integrated into the HCC-GRIm-Score (Table 1). The optimal cutoff values of NLR, AST-to-ALT ratio, and TBIL were determined by the maximally selected rank statistics using the “maxstat” package (Supplementary Figure S1). By using the exhaustive method, a HCC-GRIm-Score ≤2 points was considered as a low score.

**TABLE 1:** Univariate and multivariate Cox regression analyses of predictive factors for overall survival in training group.

Characteristics	Univariate analysis		Multivariate analysis^a^		Multivariate analysis^b^	
	HR with 95% CI	*p* value	HR with 95% CI	*p* value	HR with 95% CI	*p* value
**Clinical characteristics**						
Age (≥60 vs. <60)	0.89 (0.51–1.55)	0.670	-	-	-	-
Gender (Male vs. Female)	2.31 (1.00–5.33)	0.051	-	-	-	-
ECGO score (1–2 vs. 0)	1.05 (0.69–1.60)	0.873	-	-	-	-
Extrahepatic metastasis (present vs. absent)	1.73 (1.14–2.64)	0.011^*^	1.40 (0.79–2.46)	0.249	1.51 (0.87–2.64)	0.145
Vascular invasion (present vs. absent)	1.59 (1.03–2.44)	0.035^*^	1.17 (0.61–2.25)	0.640	1.21 (0.64–2.26)	0.560
Tumor number (multiple vs. single)	1.47 (0.94–2.31)	0.093	-	-	-	-
Maximum tumor size, cm (≥5 vs. < 5)	1.54 (0.67–3.54)	0.306	-	-	-	-
Liver cirrhosis (present vs. absent)	0.65 (0.40–1.07)	0.093	-	-	-	-
BCLC stage (stage C vs. stages A–B)	2.00 (1.16–3.45)	0.012^*^	1.40 (0.57–3.45)	0.459	1.27 (0.53–3.03)	0.598
TNM Stage (Stage III-IV vs. Stage I-II)	1.75 (1.15–2.68)	0.010^*^	-	-	-	-
Anti-HBV/HCV therapy (Yes vs. No)	0.95 (0.42–2.18)	0.905	-	-	-	-
Category of PD-1 inhibitors	1.13 (0.86–1.49)	0.396	-	-	-	-
**Original GRIm Score and the constituents**						
Original GRIm Score (high score vs. low score)	1.56 (1.23–1.99)	<0.001^*^	1.34 (1.03–1.74)	0.029^*,**^	-	-
Neutrophil-to-lymphocyte ratio (≥4.8 vs. <4.8)	1.86 (1.15–3.02)	0.012^*^	-	-	-	-
Serum albumin, g/L (<35 vs. ≥35)	2.12 (1.06–4.22)	0.034^*^	-	-	-	-
Lactate dehydrogenase, U/L (≥245 vs. < 245)	1.61 (1.04–2.49)	0.032^*^	-	-	-	-
The other blood test indicators						
PLT, 10^9^/L (≥300 vs. <300)	1.19 (0.75–1.91)	0.463	-	-	-	-
C-reactive protein, mg/L (≥8.2 vs. <8.2)	1.50 (0.95–2.37)	0.080	-	-	-	-
AST-to-ALT ratio (≥1.44 vs. <1.44)	1.86 (1.22–2.84)	0.004^*^	1.51 (0.94–2.42)	0.090^**^	-	-
Globulin, g/L (≥35 vs. <35)	1.34 (0.88–2.05)	0.171	-	-	-	-
Total bilirubin, umol/L (≥22.6 vs. <22.6)	2.15 (1.33–3.46)	0.002^*^	1.76 (1.07–2.88)	0.025^*^,^**^	-	-
*γ*-glutamyl transpeptidase,U/L (≥50 vs. <50)	2.58 (0.81–8.15)	0.107	-	-	-	-
Serum amyloid A, mg/L (≥10 vs. <10)	1.43 (0.91–2.25)	0.118	-	-	-	-
AFP, ng/ml (≥25 vs. <25)	1.10 (0.66–1.85)	0.717	-	-	-	-
CA199, U/ml (≥35 vs. <35)	1.19 (0.78–1.82)	0.429	-	-	-	-
CEA, ng/ml (≥5 vs. <5)	1.31 (0.77–2.22)	0.321	-	-	-	-
HCC-modified GRIm Score						
HCC-modified GRIm score (high score vs. low score)	–	–	–	–	2.64 (1.62–4.32)	<0.001^*^

a Multivariate regression model incorporating Original GRIm, Score.

b Multivariate regression model incorporating HCC-modified GRIm, Score.

* *p* < 0.05.

** *p* < 0.10 in the multivariate analysis and incorporated into the orignial GRIm, Score to form HCC-modified GRIm, Score.

### Follow-Up

Follow-up checkups included lab tests (tumor markers, blood routine tests, and renal and liver function) and imaging studies (enhanced CT or MRI). Patients underwent serological and imaging follow-up every 1 and 2 months after initial ICIs therapies, respectively. The last follow-up date was April 30, 2021. The primary outcome was OS, which was defined as the time interval from ICIs initiation to the date of cancer-related death or lost to follow-up.

### Statistical Analysis

Population demographics, clinical features, and tumor characteristics from training and validation groups were described as mean ± standard deviation (SD) or percentage according to the nature of the data (Table 2). Kaplan–Meier (K–M) curves were applied to estimate all time-to-event functions, and the log-rank test was calculated for *p*-value. Univariate and multivariate Cox regression analyses were performed to determine prognostic factors in terms of OS. Hazard ratios (HRs) and 95% confidence intervals (95% CIs) were also calculated. The variables with a *p* < 0.05 in the univariate analyses were inducted into the multivariate analysis using Cox proportional hazards models in the training group. The variables that has *p*-value <0.10 were integrated in the newly developed HCC-GRIm-Score. After the HCC-GRIm-Score was built, the receiver operating characteristic (ROC) curves, decision-curve analysis (DCA), and the area under the curve (AUC) were calculated based on validation group to evaluate the predictive ability among the tumor, node, and metastasis (TNM) staging system, Barcelona clinic liver cancer (BCLC) staging system, Ori-GRIm-Score, HCC-GRIm-Score, and the individual factors that included in the systems. A two-tailed *p*-value < 0.05 was considered as statistical significance. All data analyses were performed via using SPSS version 22.0 software (SPSS Inc. Chicago, IL, USA), and R version 4.0.4 statistical software (R Foundation for Statistical Computing, Vienna, Austria).

**TABLE 2:** Basic characteristic of patients in the training group, validation group and entire group.

Basic characteristics	Training group	Validation group	Entire group
Included period	2018.1–2019.12	2019.9–2020.9	2018.1–2020.9
Total number	181	80	261
Median follow-up duration (IQR), months	17.7 (12.6–22.9)	10.1 (8.2–14.8)	16.1 (9.6–20.7)
Clinical characteristics			
Age
≥60	32 (17.7%)	25 (31.3%)	57 (21.8%)
<60	149 (82.3%)	55 (68.8%)	204 (78.2%)
Gender
Male	157 (86.7%)	45 (56.3%)	202 (77.4%)
Female	24 (13.3%)	35 (43.8%)	59 (22.6%)
ECGO score
1–2	80 (44.2%)	21 (26.3%)	101 (38.7%)
0	101 (55.8%)	59 (73.8%)	160 (61.3%)
Extrahepatic metastasis
Present	78 (43.1%)	42 (52.5%)	120 (46.0%)
Absent	103 (56.9%)	38 (47.5%)	141 (54.0%)
Macrovascular invasion
Present	96 (53.0%)	44 (55.0%)	140 (53.6%)
Absent	85 (47.0%)	36 (45.0%)	121 (46.4%)
Tumor number
Single	67 (37.0%)	25 (31.3%)	92 (35.2%)
Multiple	114 (63.0%)	55 (68.8%)	169 (64.8%)
Maximum tumor size
≥5 cm	160 (90.4%)	55 (70.5%)	215 (84.3%)
<5 cm	17 (9.6%)	23 (29.5%)	40 (15.7%)
Liver cirrhosis
Present	52 (28.7%)	17 (21.3%)	69 (26.4%)
Absent	129 (71.3%)	63 (78.8%)	192 (73.6%)
Child-Pugh classification
A	0 (0%)	3 (3.8%)	3 (1.1%)
B	181 (100%)	77 (96.3%)	258 (98.9%)
BCLC Stage
Stage A–B	47 (26.0%)	14 (17.5%)	61 (23.4%)
Stage C	134 (74.0%)	66 (82.5%)	200 (76.6%)
TNM Stage
Stage I–II	96 (53.0%)	37 (46.3%)	133 (51.0%)
Stage III–IV	85 (43.0%)	43 (53.8%)	128 (49.0%)
Etiology
HBV	180 (99.4%)	76 (95.0%)	256 (98.1%)
HCV	1 (0.6%)	1 (1.3%)	2 (0.8%)
Not presented	0 (0%)	3 (3.7%)	3 (1.1%)
Etiology
Yes	167 (92.3%)	60 (75.0%)	227 (87.0%)
No	14 (7.7%)	20 (25.0%)	34 (13.0%)
Blood test indicators (±SD)			
WBC, 10^9^/L	7.55 ± 2.49	6.80 ± 2.08	7.32 ± 2.40
Neutrophil count, 10^9^/L	5.20 ± 2.24	4.44 ± 1.76	4.97 ± 2.13
Lymphocyte count, 10^9^/L	1.61 ± 0.61	1.68 ± 0.70	1.63 ± 0.64
Neutrophil-to-lymphocyte ratio	3.73 ± 2.80	3.06 ± 1.75	3.52 ± 2.54
Serum albumin, g/L	41.55 ± 4.37	42.38 ± 4.45	41.80 ± 4.41
Lactate dehydrogenase, U/L	316.2 ± 193.8	300.5 ± 230.7	311.3 ± 205.5
PLT, 10^9^/L	248.3 ± 111.7	229.6 ± 105.8	242.6 ± 110.1
C-reactive protein, mg/L	29.07 ± 39.79	16.95 ± 20.39	25.35 ± 35.41
AST, U/L	83.05 ± 79.82	74.61 ± 106.91	80.46 ± 88.87
ALT, U/L	60.38 ± 59.45	53.05 ± 44.03	58.14 ± 55.20
AST-to-ALT ratio	1.61 ± 1.09	1.41 ± 0.90	1.55 ± 1.04
Globulin, g/L	34.37 ± 5.36	36.93 ± 30.64	35.16 ± 17.54
Total bilirubin, umol/L	17.28 ± 12.17	16.97 ± 10.86	17.19 ± 11.76
*γ*-glutamyl transpeptidase, U/L	224.7 ± 254.6	190.8 ± 207.5	214.3 ± 241.2
Serum amyloid A, mg/L	62.60 ± 72.91	52.52 ± 96.95	59.51 ± 80.98
AFP, ng/ml	31947.0 ± 55330.1	16330.0 ± 34502.5	27160.2 ± 50330.8
CA199, U/ml	295.74 ± 2,107.0	348.1 ± 1982.5	311.8 ± 2065.9
CEA, ng/ml	3.73 ± 4.98	12.24 ± 67.18	6.34 ± 37.47

Abbreviations: BCLC, stage, Barcelona clinic liver cancer stage; HBV, hepatitis B virus; HCV, hepatitis C virus; WBC, white blood cells; PLT, platelets; AST, aspertate aminotransferase; ALT, alanine aminotransferase; AFP, alpha-fetoprotein; CA, carbohydrate antigen; CEA, carcinoembryonic antigen.

## Results

### Clinicopathological Characteristics of Training and Validation Groups

From January 2018 to September 2020, a total of 261 patients who received ICIs for HCC were included in the study. There were 238 (91.2%), and 23 (8.8%) patients were primary and recurrent HCC, respectively. For the recurrent HCC patients, they received surgery (N = 17) or radiofrequency ablation (N = 6) before detected recurrence and received ICIs, respectively. The medium follow-up time for the entire group was 16.10 [interquartile range (IQR), 9.60–20.70] months. For the HCC-GRIm-Score construction and validation, patients treated ICIs initially from January 2018 to December 2019 (n = 161) and from January 2020 to September 2020 (n = 80) were assigned to training and validation groups, respectively. The clinical demographics of the training and validation groups before ICIs treatment are summarized in Table 2. In terms of the overall group, patients who included in this study tended to be younger (<60 years, 78.2%), have larger tumor size (≥5 cm, 84.3%), have higher percentages of absence of cirrhosis (73.6%), at more advanced stage (BCLC C stage, 76.6%), and higher hepatitis infectious rates (87.0%). There were no significant differences in terms of clinicopathological characteristics between the training and validation groups (Table 2).

### Development and Evaluation of the HCC-GRIm-Score System

The potential prognostic factors including basic characteristics, liver function, tumor burden, and tumor markers were included in the univariate analysis for OS. As results, the significant factors that were identified from univariate analysis were introduced to multivariate analysis, including extrahepatic metastasis (*p* = 0.011), vascular invasion (*p* = 0.035), BCLC staging (*p* = 0.012), Ori-GRIm-Score (*p* < 0.001), NLR (*p* = 0.012), ALB (*p* = 0.034), LDH (*p* = 0.032), AST-to-ALT ratio (*p* = 0.004), and TBIL (*p* = 0.002). The following multivariate analysis indicated the Ori-GRIm-Score (*p* = 0.029), AST-to-ALT ratio (*p* = 0.090), and TBIL (*p* = 0.025) as independent prognostic factors for OS (*p* < 0.100; Table 1).

**FIGURE 1:**
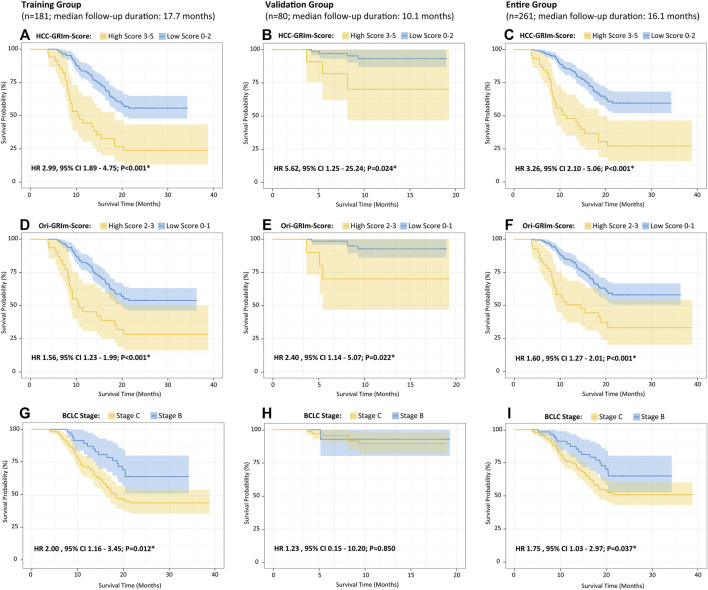
Kaplan–Meier curves of the overall survival for HCC patients after ICIs therapies divided by HCC-GRIm-Score **(A–C)**, Ori-GRIm-Score **(D–F)**, and BCLC staging system **(G–I)** in terms of training group, validate group, and entire group, respectively. HCC, hepatocellular carcinoma; ICIs, immune checkpoint inhibitors; BCLC, Barcelona clinic liver cancer stage; HR, hazard ratio; CI, confidence interval.

The identified independent prognostic factors that were mentioned above were integrated to generate the HCC-GRIm-Score. The HCC-GRIm-Score was constructed via using ALB (<35 g/L = 1), LDH (>245 U/L = 1), NLR (≥4.8 = 1), AST-to-ALT ratio (≥1.44 = 1), and TBIL (≥22.6 umol/L = 1). According to the HCC-GRIm-Score, patients in the training group with a low score (0, 1, or 2 points) had a significantly longer median OS of not reached (NR) than patients with a high score (3, 4, or five points) who had a median OS of 10.3 months (HR, 2.99; 95% CI, 1.89–4.75; *p* < 0.001; Figure 1A).

**FIGURE 2:**
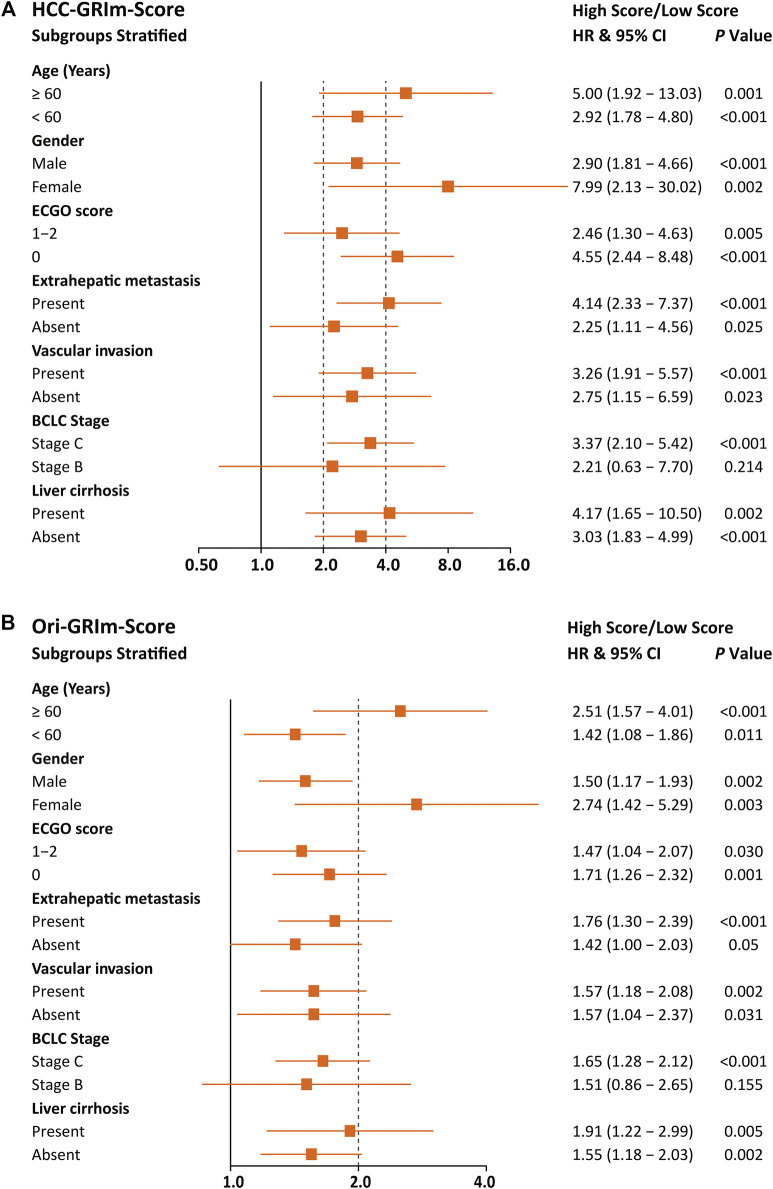
Forest plots stratified by HCC-GRIm-Score **(A)** and Ori-GRIm-Score **(B)**. HCC, hepatocellular carcinoma; HR, hazard ratio; CI, confidence interval.

To further evaluate the improved HCC-GRIm-Score and Ori-GRIm-Score, the multivariate analysis of subgroups stratified forest plots based on the entire group were drawn to investigate the stability and consistency in separating the risk population from low score against the high score for different factors (Figure 2). The forest plot demonstrated that both HCC-GRIm-Score and Ori-GRIm-Score showed reliable discriminatory power to identify the high-score population as high risk for adverse prognosis over low-score population (HR > 1.000, *p* < 0.050).

### Comparison of Ori-GRIm-Score, HCC-GIRm-Score, TNM and BCLC

**FIGURE 3:**
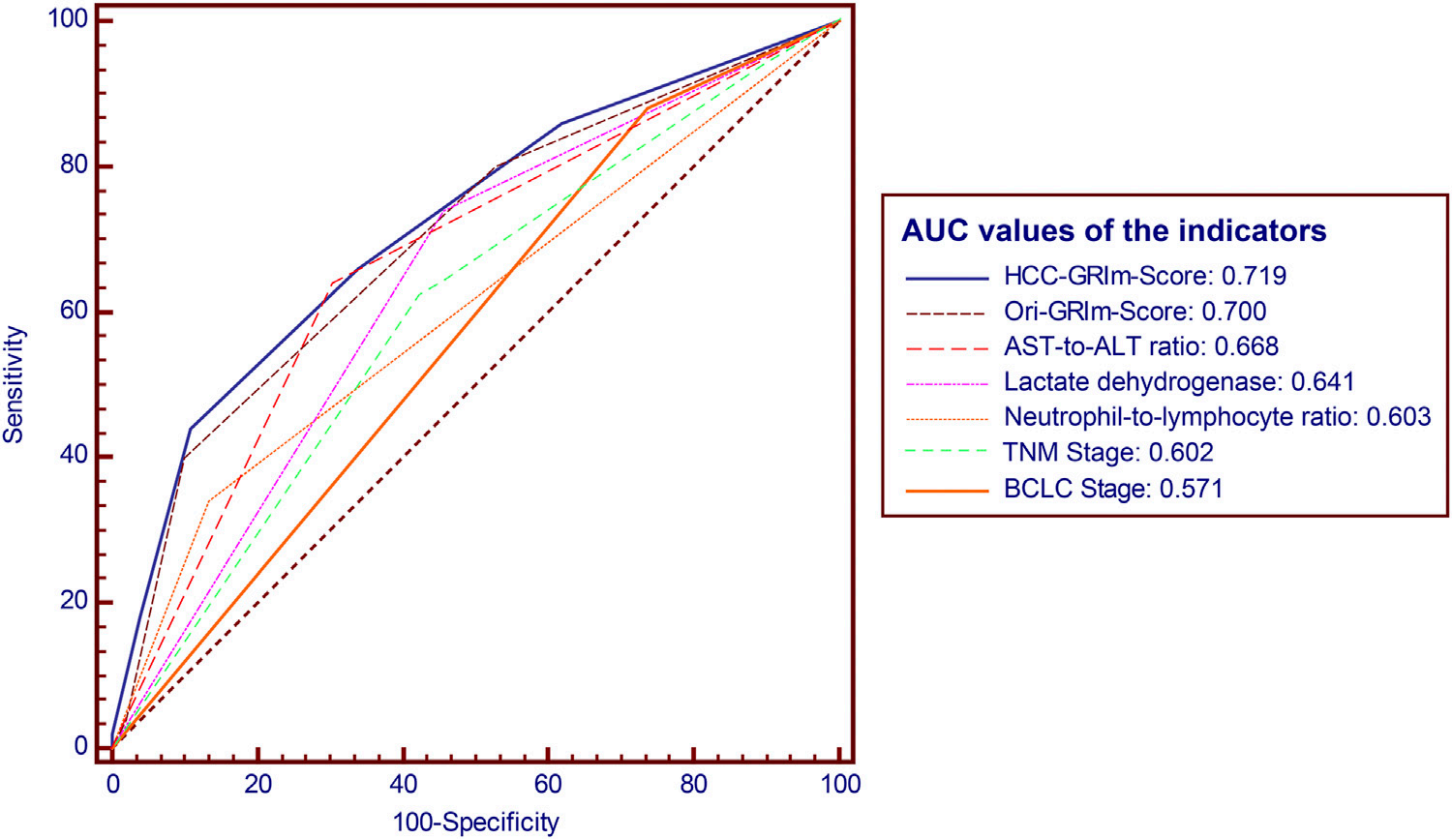
ROC and AUC for survival prediction of the enrolled scoring systems. ROC, receiver operating characteristic; AUC, area under curve; HCC, hepatocellular carcinoma, AST, aspartate aminotransferase; ALT, alanine aminotransferase; BCLC, Barcelona clinic liver cancer stage.

**FIGURE 4:**
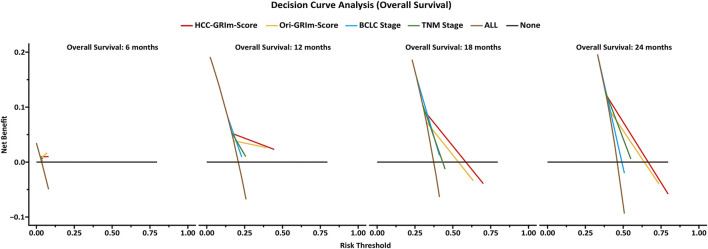
Decision-curve analysis (DCA) plot depicting the standardized net benefit among HCC-GRIm-Score, Ori-GRIm-Score, TNM, and BCLC staging system at 6, 12, 18, and 24 months. HCC, hepatocellular carcinoma; TNM, tumor, node and metastasis; BCLC, Barcelona clinic liver cancer.

The predictive accuracy and probability among Ori-GRIm-Score, HCC-GRIm-Score, TNM, and BCLC staging system were compared *via* ROC and DCA. ROC curves for the 1-year OS were plotted based on the 80 patients from the validation group. The discriminatory ability of the HCC-GRIm-Score, which had a C-index corresponding to the area under the ROC curve of 0.719 (95% CI, 0.661–0.773), was superior to that of the Ori-GRIm-Score, TNM, and the BCLC staging system with AUC of 0.700 (95% CI, 0.640–0.755), 0.602 (95% CI, 0.540–0.662), and 0.571 (95% CI, 0.508–0.632), respectively (Figure 3). The DCA curves were also depicted with respect to 6-, 12-, 18-, and 24-month OS. The DCA curves preliminarily indicated that HCC-GRIm-Score gave better net benefit in the prediction of 12-, 18-, and 24-month OS compared to those of Ori-GRIm-Score, TNM, and BCLC staging system (Figure 4).

Moreover, when applying the HCC-GRIm-Score to the validation group (HR, 5.62; 95% CI, 1.25–25.24; *p* = 0.024) and entire group (HR 3.26, 95% CI, 2.10–5.06; *p* < 0.001), respectively, it can still distinguish different OS between high and low score significantly (Figures 1B,C). The K–M curves of OS from Ori-GRIm-Score and the BCLC staging system based on training, validation, and entire groups were also plotted (Figures 1D–I).

## Discussion

In the present study, we firstly applied, modified, and validated the Ori-GRIm-Score system based on HCC patients treated with ICIs. The optimized HCC-GRIm-Score integrated ALB, LDH, NLR, AST-to-ALT ratio and TBIL indicated that HCC patients treated by ICIs with high scores (HCC-GRIm-Score>2 points) suffered from adverse prognosis, and it also demonstrated superior prognostication performance compared to the Ori-GRIm-Score, TNM staging system and BCLC staging system (AUC, 0.719 vs. 0.700 vs. 0.602 vs. 0.571, respectively).

Although the introduction of ICIs into the HCC treatment has improved the survival of patients in recent years, the response rates of ICIs were still limited because of the variable immune conditions of patients (Zhou ZG et al., 2020; Schoenfeld and Hellmann, 2020; Pan et al., 2021). Unfortunately, there is no biological marker available in predicting the responses to ICIs regarding HCC. Moreover, the BCLC staging system and TNM staging system had their limitations in predicting HCC patients who received ICIs therapies in the clinical practice (Trovato et al., 2013; Yang et al., 2015). Therefore, it is valuable to develop new tools to identify the HCC patients who have high probability to benefit from ICIs treatment. Moreover, based on the multivariable analysis of present study, the traditional high-risk factors for OS, such as tumor size, liver cirrhosis, and tumor markers, were no longer independent prognostic factors for HCC patients who received ICIs therapies. Recently, serials of scoring systems were carried out and presented promising ability in prediction of cancer prognosis (Grenader et al., 2016; Zhao et al., 2016; Chan et al., 2017; Okugawa et al., 2020). Based on the encouraging results, oncologists further applied these evaluation systems to patients who underwent ICIs therapies (Dharmapuri et al., 2020). Ori-GRIm-Score is a classical scoring system proposed ALB, LDH, and NLR in reflecting immune system status, and it provided a better selection of patients in treating different cancers with ICIs (Bigot et al., 2017; Lenci et al., 2021). In order to adapt the Ori-GRIm-Score for HCC, two liver-specific parameters, AST-to-ALT ratio and TBIL, were identified and integrated into the HCC-GRIm-Score. According to previous experience, the decreased ALB, increased LDH or increased NLR has been proved to be independent negative prognostic factors for OS (Arkenau et al., 2008; Bigot et al., 2017). The AST-to-ALT ratio was reported to stratify HBV-related HCC patients with distinguishable prognosis effectively after hepatectomy (Shen et al., 2021). And the TBIL was also found as a prognostic factor for OS in terms of HCC patients previously (Yang et al., 2020). However, according to the MD Anderson experience, hemoglobin level, under 105 g/L, was also an independent factor for poor survival as well as performance status and tumor types (Garrido-Laguna et al., 2012). Those factors were not prognostic in the above multivariate mode; thus, they were not used in the HCC-GRIm-Score.

Since liver function is a crucial aspect of long-term prognosis especially for advanced HCC patients (Lo et al., 2017; Zhao et al., 2020), the current HCC-GRIm-Score took liver profiles into consideration which improved the predictive ability of the Ori-GRIm-Score for HCC patients in the treatment of ICIs. Notably, by using the GRIm-Score, there were 10 and six patients in the validation group who were exchanged from low score to high score or vias versa, respectively. As a result, the median OS of low and high scores in the entire group were improved from NR vs. 11.4 (HR, 1.60; 95% Cl; 127–2.01; *p* < 0.001; Figure 1F) to NR vs. 10.6 (HR 3.26, 95% CI 2.10-5.06; *p* < 0.001; Figure 1C) according to Ori-GRIm-Score and HCC-GRIm-Score, respectively. Similar results were observed in the validation and entire groups, respectively. Therefore, the discriminatory power of the Ori-GRIm-Score was further optimized. The similar outcomes were also found in comparison to the BCLC staging system (Figure 1I). Additionally, the subgroups-stratified multivariable analysis was conducted to reevaluate the discriminatory power of Ori-GRIm-Score and HCC-GRIm-Score for other prognostic factors in HCC patients who received ICIs, which again confirmed the findings (Figure 2). Interestingly, when further investigating the clinical features of the patients who were stratified by Ori-GRIm-Score or HCC-GRIm-Score, the presence of macrovascular invasion had significantly higher percentages in the high score group (Supplementary Table S2). It indicated that the GRIm-Score based scoring systems showed good ability in discriminating HCC patients with potential vascular invasion, and the mechanism behind this needs to be further studied in the future.

The clinical factors that were mentioned in the present study have been validated separately in previous studies (Chan et al., 2015; Wang et al., 2018; Liao et al., 2021). To the best of our knowledge, the present study is the first to combine them together to assess patients who are subjected to ICIs therapies. Hence, the proposed HCC-GRIm-Score can be used as a reference to making treatment strategies towards ICIs. The high-intensity treatment strategies should be warranted when patients were characterized with a high score based on the HCC-GRIm-Score. The high-end imaging examinations and close follow-up could also be counseled to receive more. Conversely, the treatment strategy and follow-up period for the low score patients could be mild and priority to safety. Patients with low HCC-GRIm-Score showed satisfactory treatment effects to ICIs, the present modalities could be adopted. However, for high score patients, other treatment options should be considered.

The present study has some limitations. First, it is a retrospective study with a restricted case volumes based on a single-center cohort in China. Hence, the results from current study need to be validated in larger population, ideally prospective and multicenter trials. Second, most patients received combination therapies that included different types of ICIs during the whole treatment, which inevitably caused bias. Third, given the majority of the included patients had an HBV background, which was considered as the main etiology for HCC, the extrapolation of the current results should be cautious if the HCCs were thought to be caused by hepatitis C virus infection, alcohol abuse, or obesity.

## Conclusion

We developed and validated the HCC-GRIm-Score system that integrated ALB, LDH, NLR, AST-to-ALT ratio, and TBIL based on HCC patients who received ICIs therapies. The discriminatory power of HCC-GRIm-Score was optimized when liver profiles were taken into account. The present HCC-GRIm-Score system needs to be further validated in a large, multicenter dataset in the future.

## Data Availability

The raw data supporting the conclusion of this article will be made available by the authors, without undue reservation.

## Abbreviations

BCLC stage, Barcelona clinic liver cancer stage; HBV, hepatitis B virus; HCV, hepatitis C virus; WBC, white blood cells; PLT, platelets; AST, aspertate aminotransferase; ALT, alanine aminotransferase; AFP, alpha-fetoprotein; CA, carbohydrate antigen; CEA, carcinoembryonic antigen.
