# Supplementary material for: Development and Validation of a Prognostic Score for Hepatocellular Carcinoma Patients in Immune Checkpoint Inhibitors Therapies: The Hepatocellular Carcinoma Modified Gustave Roussy Immune Score
Source: Front Pharmacol. 2022 Feb 8;12:819985. doi: 10.3389/fphar.2021.819985 (PMC8883391; doi:10.3389/fphar.2021.819985)
Supplement: Supplementary file 1 [file Table1.DOCX]

**Table S1** Category and dosage of PD-1 inhibitors used in the patients.

| **Category** | **Dose (mg)** | **Training group** | **Validation group** | **Entire group** |
| --- | --- | --- | --- | --- |
| Pembrolizumab  (Carlow, Merck Sharp & Dohme Corp.) | 200 | 15 (8.3%) | 25 (31.3%) | 40 (15.3%) |
| Nivolumab  (Bristol-Myers Squibb) | 100 | 2 (1.1%) | 3 (3.8%) | 5 (1.9%) |
| Toripalimab  (Suzhou, hezhong pharmaceutical Co. Ltd) | 240 | 115 (63.5%) | 13 (16.3%) | 128 (49.0%) |
| Sintilimab  (Suzhou, xinda pharmaceutical Co. Ltd) | 200 | 44 (24.3%) | 23 (28.7%) | 67 (25.7%) |
| Tislelizumab  (Boehringer-Ingelheim pharmaceutical Co. Ltd) | 200 | 0 (0%) | 4 (5.0%) | 4 (1.5%) |
| Camrelizumab  (Suzhou, shengdiya pharmaceutical Co. Ltd) | 200 | 5 (2.8%) | 12 (15.0%) | 17 (6.5%) |
