# Supplementary material for: Development and Validation of a Prognostic Score for Hepatocellular Carcinoma Patients in Immune Checkpoint Inhibitors Therapies: The Hepatocellular Carcinoma Modified Gustave Roussy Immune Score
Source: Front Pharmacol. 2022 Feb 8;12:819985. doi: 10.3389/fphar.2021.819985 (PMC8883391; doi:10.3389/fphar.2021.819985)
Supplement: Supplementary file 2 [file Table2.DOCX]

**Table S2** Clinical characteristics of patients in the training group and validation group classified by original GRIm scores and HCC-modified GRIm scores.

|  | **Original GRIm Score** | | | | | |  | **HCC-modified GRIm Score** | | | | | |
| --- | --- | --- | --- | --- | --- | --- | --- | --- | --- | --- | --- | --- | --- |
|  | Training Group | | *P* value | Validation Group | | *P* value |  | Training Group | | *P* value | Validation Group | | *P* value |
|  | Low Score | High Score |  | Low Score | High Score |  |  | Low Score | High Score |  | Low Score | High Score |  |
| **Clinical characteristics** |  |  |  |  |  |  |  |  |  |  |  |  |  |
| Age |  |  | 0.385 |  |  | 0.927 |  |  |  | 0.564 |  |  | 0.511 |
| ≥ 60 | 24 | 7 |  | 22 | 3 |  |  | 24 | 7 |  | 23 | 2 |  |
| < 60 | 125 | 24 |  | 48 | 7 |  |  | 122 | 27 |  | 46 | 9 |  |
| Gender |  |  | 0.169 |  |  | 0.201 |  |  |  | 0.588 |  |  | 0.838 |
| Male | 132 | 24 |  | 37 | 8 |  |  | 128 | 28 |  | 38 | 7 |  |
| Female | 17 | 7 |  | 33 | 2 |  |  | 18 | 6 |  | 31 | 4 |  |
| ECGO score |  |  | 0.579 |  |  | 0.923 |  |  |  | 0.057 |  |  | 0.651 |
| 1-2 | 64 | 15 |  | 19 | 2 |  |  | 59 | 20 |  | 17 | 4 |  |
| 0 | 85 | 16 |  | 51 | 8 |  |  | 87 | 14 |  | 52 | 7 |  |
| Extrahepatic metastasis |  |  | 0.488 |  |  | 0.612 |  |  |  | 0.184 |  |  | 0.884 |
| Present | 62 | 15 |  | 38 | 4 |  |  | 59 | 18 |  | 36 | 6 |  |
| Absent | 87 | 16 |  | 32 | 6 |  |  | 87 | 16 |  | 33 | 5 |  |
| Macrovascular invasion |  |  | 0.026* |  |  | 0.497 |  |  |  | 0.002* |  |  | 0.024* |
| Present | 73 | 22 |  | 37 | 7 |  |  | 69 | 26 |  | 34 | 10 |  |
| Absent | 76 | 9 |  | 33 | 3 |  |  | 77 | 8 |  | 35 | 1 |  |
| Tumor number |  |  | 0.530 |  |  | 0.649 |  |  |  | 0.514 |  |  | 0.965 |
| Single | 57 | 10 |  | 23 | 2 |  |  | 56 | 11 |  | 21 | 4 |  |
| Multiple | 92 | 21 |  | 47 | 8 |  |  | 90 | 23 |  | 48 | 7 |  |
| Tumor size |  |  | 0.827 |  |  | 0.069 |  |  |  | 0.695 |  |  | 0.050 |
| ≥ 5 cm | 131 | 29 |  | 45 | 10 |  |  | 128 | 32 |  | 44 | 11 |  |
| < 5 cm | 14 | 2 |  | 23 | 0 |  |  | 14 | 2 |  | 23 | 0 |  |
| Liver cirrhosis |  |  | 0.373 |  |  | 0.757 |  |  |  | 0.940 |  |  | 0.999 |
| Present | 41 | 11 |  | 14 | 3 |  |  | 42 | 10 |  | 15 | 2 |  |
| Absent | 108 | 20 |  | 56 | 7 |  |  | 104 | 24 |  | 54 | 9 |  |
| Child-Pugh classification |  |  | – |  |  | 0.366 |  |  |  | – |  |  | 0.481 |
| A | 0 | 0 |  | 3 | 0 |  |  | 0 | 0 |  | 3 | 0 |  |
| B | 149 | 31 |  | 67 | 10 |  |  | 146 | 34 |  | 66 | 11 |  |
| BCLC Stage |  |  | 0.347 |  |  | 0.505 |  |  |  | 0.093 |  |  | 0.717 |
| Stage A-B | 41 | 6 |  | 11 | 3 |  |  | 42 | 5 |  | 13 | 1 |  |
| Stage C | 108 | 25 |  | 59 | 7 |  |  | 104 | 29 |  | 56 | 10 |  |
| TNM Stage |  |  | 0.316 |  |  | 0.351 |  |  |  | 0.232 |  |  | 0.955 |
| Stage I-II | 82 | 14 |  | 31 | 6 |  |  | 81 | 15 |  | 32 | 5 |  |
| Stage III-IV | 67 | 17 |  | 39 | 4 |  |  | 65 | 19 |  | 37 | 6 |  |
| Etiology |  |  | 0.538 |  |  | 0.705 |  |  |  | 0.517 |  |  | 0.690 |
| HBV | 148 | 31 |  | 66 | 10 |  |  | 145 | 34 |  | 65 | 11 |  |
| HCV | 1 | 0 |  | 1 | 0 |  |  | 1 | 0 |  | 1 | 0 |  |
| Not presented | 0 | 0 |  | 3 | 0 |  |  | 0 | 0 |  | 3 | 0 |  |
| Anti-HBV/HCV therapy |  |  | 0.999 |  |  | 0.999 |  |  |  | 0.918 |  |  | 0.851 |
| Yes | 137 | 29 |  | 52 | 8 |  |  | 134 | 32 |  | 51 | 9 |  |
| No | 12 | 2 |  | 18 | 2 |  |  | 12 | 2 |  | 18 | 2 |  |
| **Original GRIm Score Constituents** | |  |  |  |  |  |  |  |  |  |  |  |  |
| Neutrophil-to-lymphocyte ratio |  |  | <0.001* |  |  | <0.001* |  |  |  | <0.001* |  |  | <0.001* |
| < 4.8 | 140 | 6 |  | 66 | 3 |  |  | 135 | 11 |  | 64 | 5 |  |
| ≥ 4.8 | 9 | 25 |  | 4 | 7 |  |  | 11 | 23 |  | 5 | 6 |  |
| Serum albumin, g/L |  |  | <0.001* |  |  | <0.001* |  |  |  | <0.001* |  |  | 0.004* |
| ≥ 35 g/L | 146 | 21 |  | 69 | 4 |  |  | 143 | 24 |  | 66 | 7 |  |
| < 35 g/L | 3 | 10 |  | 1 | 6 |  |  | 3 | 10 |  | 3 | 4 |  |
| Lactate dehydrogenase |  |  | <0.001* |  |  | <0.001* |  |  |  | <0.001* |  |  | <0.001* |
| < 245 U/L | 80 | 1 |  | 46 | 0 |  |  | 79 | 2 |  | 46 | 0 |  |
| ≥ 245 U/L | 69 | 30 |  | 24 | 10 |  |  | 67 | 32 |  | 23 | 11 |  |

Abbreviations: BCLC stage, Barcelona clinic liver cancer stage; HBV, hepatitis B virus; HCV, hepatitis C virus.

**P*<0.05.
