# Supplementary material for: Development and Validation of a Prognostic Score for Hepatocellular Carcinoma Patients in Immune Checkpoint Inhibitors Therapies: The Hepatocellular Carcinoma Modified Gustave Roussy Immune Score
Source: Front Pharmacol. 2022 Feb 8;12:819985. doi: 10.3389/fphar.2021.819985 (PMC8883391; doi:10.3389/fphar.2021.819985)
Supplement: Supplementary file 3 [file DataSheet1.PDF]

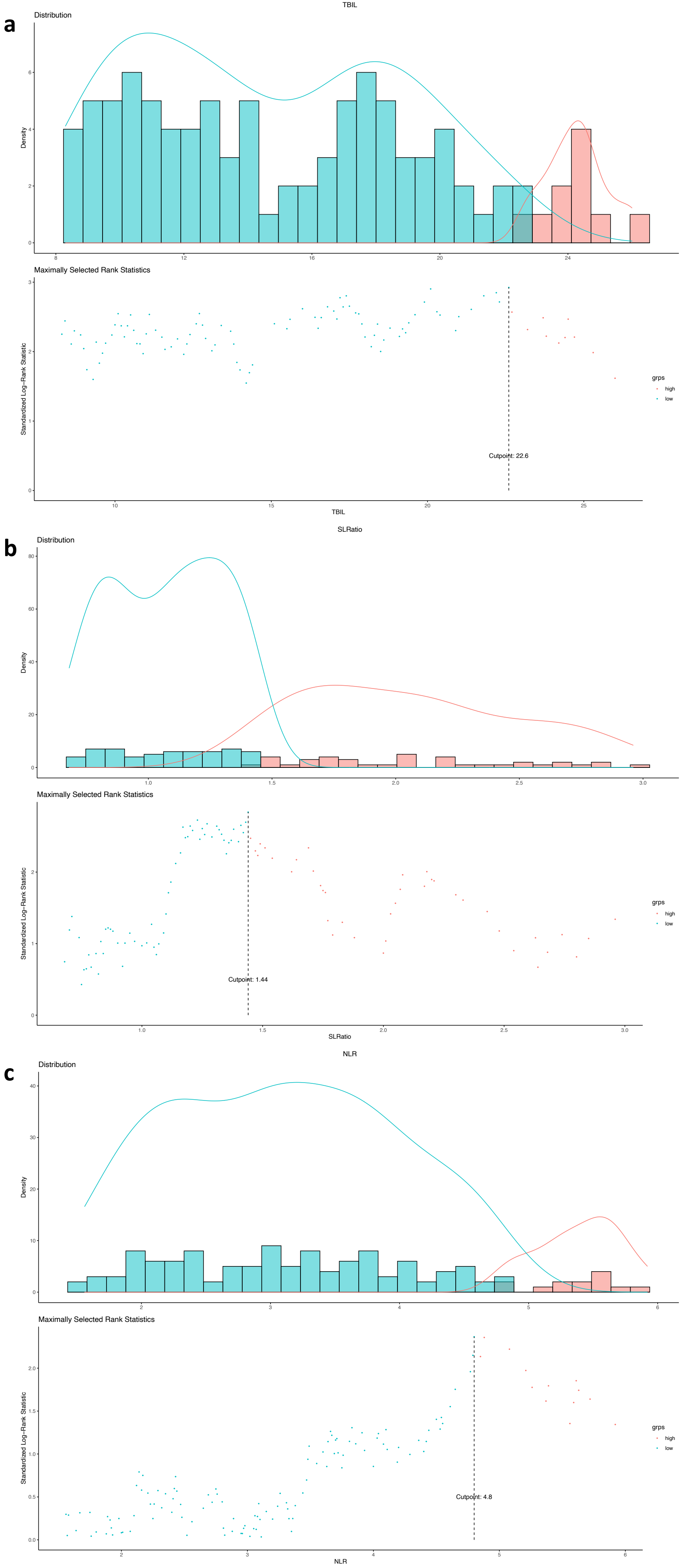

Figure S1 Schematic of the method used to determine the optimal cutoff points of total bilirubin, aspartate transaminase-to-alanine transaminase ratio, neutrophil to lymphocyte ratio using the "maxstat" package. TBIL, total bilirubin; SLRation, aspartate transaminase-to-alanine transaminase ratio; NLR, neutrophil to lymphocyte ratio.
